# P01.27. Assessment of polyphenols on PSA expression in a human co-culture model of reactive prostate stroma cells and LAPC4 prostate adenocarcinoma cells

**DOI:** 10.1186/1472-6882-12-S1-P27

**Published:** 2012-06-12

**Authors:** G Vollmer, H Amri, J Arnold

**Affiliations:** 1 Technical University Dresden, Dresden, Germany; 2 Georgetown University Medical Center, Washington, D.C., USA; 3 NCCAM/NIH, Bethesda, USA

## Purpose

The reactive stromal phenotype in the prostate is mechanistically important for understanding prostate cancer progression and may be a target for prevention. We mimicked the interaction of endocrine, paracrine, and immune factors on induced androgen metabolism in prostate stroma by coculturing human primary prostate stromal cells and LAPC-4 prostatic adenocarcinoma cells. The aim of the study was to use this model to investigate dose dependent effects of hop-derived polyphenols xanthohumol (Xan), isoxanthohumol (iXan), 8-prenylnaringenin (8-PN) and structurally-related compounds quercetin (Que), naringenin (Nar), and 6-dimethylallylnaringenin (6-DMAN) on PSA secretion.

## Methods

Conversion of DHEA (D) by the reactive stroma to androgens following TGF-beta (T) stimulation was assessed by induced PSA secretion in LAPC-4 cells. Direct epithelial PSA secretion was induced using the non-metabolizable androgen R1881. The natural compounds were used in doses of 0.01-10 µM (Nar, 8-PN, 6-DMAN) or 0.1-10 µM (Xan, iXan, Que).

## Results

All compounds tested dose-dependently attenuated epithelial PSA production resulting from stromal androgen metabolite production following D+T treatment (stromal-mediated response). Following direct epithelial stimulation by R1881 treatment (‘epithelial response’), 8-PN and 6-DMAN also showed a dose-dependent response pattern. All other compounds inhibited this epithelial response only at the highest dose (10 µM). Comparative studies with pure estrogen receptor agonists demonstrated the involvement of these receptors in mediating the response, however the high inhibition of the stromal response by Que, Xan, iXan and Nar cannot be explained by ER-dependent mechanisms only, as they are very weak estrogens.

## Conclusion

In conclusion, hop-derived polyphenols, as well as structurally related compounds are very potent inhibitors of stromal conversion of androgenic prohormones. Only the two most potent estrogenic compounds showed the same inhibition of PSA induction in response to R1881 treatment. More studies are needed to examine the value of these compounds in prevention of prostate cancer progression.

